# Computational Biology Helps Understand How Polyploid Giant Cancer Cells Drive Tumor Success

**DOI:** 10.3390/genes14040801

**Published:** 2023-03-26

**Authors:** Matheus Correia Casotti, Débora Dummer Meira, Aléxia Stefani Siqueira Zetum, Bruno Cancian de Araújo, Danielle Ribeiro Campos da Silva, Eldamária de Vargas Wolfgramm dos Santos, Fernanda Mariano Garcia, Flávia de Paula, Gabriel Mendonça Santana, Luana Santos Louro, Lyvia Neves Rebello Alves, Raquel Furlani Rocon Braga, Raquel Silva dos Reis Trabach, Sara Santos Bernardes, Thomas Erik Santos Louro, Eduardo Cremonese Filippi Chiela, Guido Lenz, Elizeu Fagundes de Carvalho, Iúri Drumond Louro

**Affiliations:** 1 Centro de Ciências Humanas e Naturais, Departamento de Ciências Biológicas, Universidade Federal do Espírito Santo (UFES), Vitória 29075-910, Brazil; matheus.c.casotti@gmail.com (M.C.C.);; 2 Centro de Ciências da Saúde, Curso de Medicina, Universidade Federal do Espírito Santo (UFES), Vitória 29090-040, Brazil; 3 Departamento de Patologia, Instituto de Ciências Biológicas, Universidade Federal de Minas Gerais (UFMG), Belo Horizonte 31270-901, Brazil; 4 Escola Superior de Ciências da Santa Casa de Misericórdia de Vitória (EMESCAM), Vitória 29027-502, Brazil; 5 Departamento de Ciências Morfológicas, Instituto de Ciências Básicas da Saúde, Universidade Federal do Rio Grande do Sul (UFRGS), Porto Alegre 90035-003, Brazil; 6 Serviço de Pesquisa Experimental, Hospital de Clínicas de Porto Alegre, Porto Alegre 90035-903, Brazil; 7 Centro de Biotecnologia, Universidade Federal do Rio Grande do Sul, Porto Alegre 91501-970, Brazil; 8 Departamento de Biofísica, Instituto de Biociências, Universidade Federal do Rio Grande do Sul (UFRGS), Porto Alegre 91501-970, Brazil; 9 Instituto de Biologia Roberto Alcântara Gomes (IBRAG), Universidade do Estado do Rio de Janeiro (UERJ), Rio de Janeiro 20551-030, Brazil

**Keywords:** polyploid giant cancer cells (PGCCs), bioinformatics, systems biology, tumor evolution

## Abstract

Precision and organization govern the cell cycle, ensuring normal proliferation. However, some cells may undergo abnormal cell divisions (neosis) or variations of mitotic cycles (endopolyploidy). Consequently, the formation of polyploid giant cancer cells (PGCCs), critical for tumor survival, resistance, and immortalization, can occur. Newly formed cells end up accessing numerous multicellular and unicellular programs that enable metastasis, drug resistance, tumor recurrence, and self-renewal or diverse clone formation. An integrative literature review was carried out, searching articles in several sites, including: PUBMED, NCBI-PMC, and Google Academic, published in English, indexed in referenced databases and without a publication time filter, but prioritizing articles from the last 3 years, to answer the following questions: (i) “What is the current knowledge about polyploidy in tumors?”; (ii) “What are the applications of computational studies for the understanding of cancer polyploidy?”; and (iii) “How do PGCCs contribute to tumorigenesis?”

## 1. Introduction

The cell cycle is a series of events that occur to accurately replicate genetic material and cellular contents into daughter cells. This regulation is precisely monitored and controlled [1]. Nevertheless, some cells can undergo alternative cellular divisions or mitotic cycle variations, generating aneuploid (polyploid) genomes in cells with varying cell sizes [1].

Polyploidy, a cell that has three or more chromosomal sets [2], in cancer is complex, primarily because the initiation and progression of cancer involves multiple integrated processes [3]. The relationship between PGCCs (cell subpopulation with a chromosome content greater than 2n) and cancer has recently reached interesting results regarding the fundamental role of these cells for tumor survival, immortalization, aggressiveness, and progression [4].

Fei et al. [3], Glassmann et al. [5], and Fei et al. [6] showed the presence of PGCCs in breast, colorectal, and ovarian cancer, correlating with poor survival and prognosis. These cells are considered an intermediate product of genomic instability [7].

Recent data demonstrated that PGCCs may form from oncogenic and therapeutic stress, generating reprogrammed cancer cells [8] and being a source of cancer stem cells (CSC) [9], influencing tumor resistance [10], and auto renewal [11]. PGCCs exhibit elevated aneuploidy and are connected to the tumor microenvironment evolution [12]; they have a cell cycle of their own, called “giant cell cycle” [1]. They are also able to: (i) create or repopulate macroscopic spheroids in vitro [13], (ii) generate tumors when inoculated in mice [14], and (iii) convert into different phenotypes, showing high plasticity [15], which has been included as a new hallmark of cancer.

Bharadwaj et al. [16], Mirzayans, Andrais, and Murray [17], Mirzayans and Murray [18,19], and Bharadwaj and Mandal [20] showed the connection between senescence and PGCCs, as cancer cells can escape from premature senescence, possibly leading to the formation of a multinucleated gigantic cell. Zhang et al. [21,22], Lin et al. [23], Sirois et al. [24], White-Gilbertson et al. [25], and Voelkel-Johnson [26] highlighted the metabolic and biophysical aspects of PGCCs, including cytoskeleton and biochemical modifications, that sustain cell cycle dysregulation, stress responses, and dedifferentiation.

Numerous studies have begun to use high throughput approaches through multiple techniques, including next generation sequencing (NGS), multi-omics, and phylostratigraphy, among others. Additionally, this review seeks to integrate theories, show different hypotheses, emphasize the need for multidisciplinary and collaborative approaches, demonstrate possible therapeutic options, and highlight the impact of ploidy on different stages of carcinogenesis. Our goal is to discuss the following questions: (i) “What is the current knowledge about polyploidy in tumors?”; (ii) “What are the applications of computational studies for the understanding of cancer polyploidy?”; and (iii) “How do PGCCs contribute to tumorigenesis?”

## 2. Polyploidy in Cancer

Cell cycle deregulation and/or failures in mitosis can lead to polyploidy, the upregulation of genes that positively impact the cell cycle progression, and cytostatic genes downregulation [27,28] in physiological and pathological manners [29]. Polyploidy, or whole genome duplication (WGDs), results from the cell cycle prematurely ending or from cellular fusion [30], increasing genetic variation, stress tolerance, the ability to colonize different environments, and mutation burden relief. The evolutionary selective advantages of WGD offer a theoretical base for cyclic ploidy, mitotic slippage, and cancer cell fusion [31].

Polyploid cells are vulnerable during meiosis due to chromosome pairing difficulties and genomic instability [30]; this can be solved by a transient reversion of polyploidy (depolyploidization) [32], multipolar division [33], chromosomal rearrangement, and DNA divergence. A high degree of polyploidy or aneuploidy are detrimental to the cell, but can increase cellular adaptability and plasticity, fueling intratumoral heterogeneity [34,35].

## 3. A Brief Introduction to PGCCs

PGCCs have been observed for at least 180 years in diverse cancer types [36,37,38,39]. Some were observed after chemotherapy, in cells with irregular nuclei, increased migratory potential, genetic instability, altered response to hypoxia, drug resistance, or higher mortality rates [40].

PGCCs are a special subpopulation of tumor cells containing a large cytoplasm and multiple nuclei [41], undergoing an altered giant cell cycle, creating genetic heterogeneity, and allowing cancer cells to overcome multiple micro-environment challenges [42].

### 3.1. PGCC’s Giant Cell Cycle

Stress factors such as chemotherapy, antimitotic drugs, radiotherapy, hypoxia, or a deficient microenvironment may induce PGCCs formation [28] by means of a giant cell cycle (cycle linked to polyploidization and depolyploidization processes together with neosis) used for dedifferentiation of somatic cells, which can generate stem cells for tumor initiation. Such a cell cycle is divided in four phases: (i) Initiation: stressed tumor cells undergo catastrophic mitosis or cell death. Surviving cells assume a tetraploid or polyploid transient state [1,11]; (ii) Auto-renovation: tetraploid cells start endocycling to produce mononuclear or multinuclear PGCCs. Some multinuclear cells go through cyto-fission to create smaller polyploid cells [1,11]; (iii) Termination: PGCCs undergo depolyploidization (genome reduction division) and generate diploid nuclei by budding. Others form a structure that resembles a reproductive cyst [1,11]; and (iv) Stability: diploid descendent cells with altered genotypes continue to differentiate into a variety of aneuploid cell types with proliferative capacity [1,11].

#### Giant Cell Cycle Possible Outcomes and Fates

**Figure 1:**
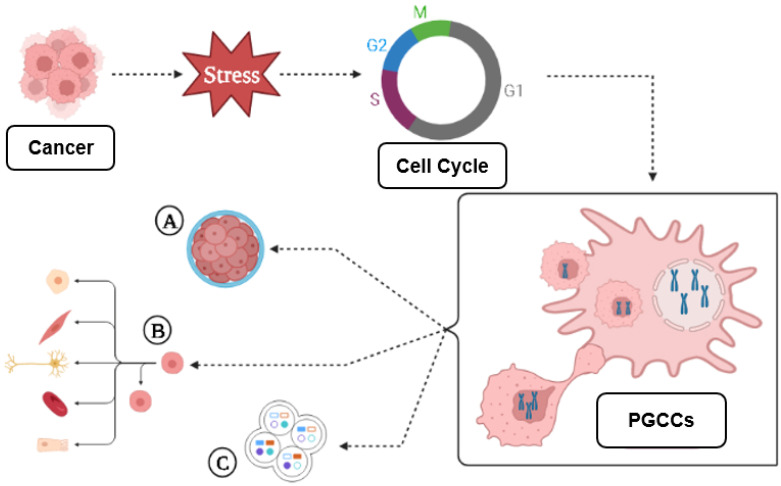
Giant cell cycle outcomes. Under stress, the cell responds in various ways to maintain balance. When chemotherapy treated tumor cells are isolated, large numbers of cells die; surviving cells may start an altered cell cycle, capable of generating PGCCs, which follow four phases or other unusual divisions; PGCCs exhibit abnormal shapes similar to single-celled organisms such as amoebas, or embryonic cells at the blastomere stage; that (A) can undergo diapause, dormancy, relapse, and generate multilineages; (B) tumor cells use these means to survive and generate resistant daughter cells by budding, fragmentation (bursting), sporulation or encystment; (C) new genetic and epigenetic characteristics create unlimited potential for more aggressive, resistant and immortal phenotypes. Terms are shown in Box A1 (Appendix A). Note: created with BioRender.com (accessed on 15 March 2023).

There is a correlation between endoreplication cell cycles and the amount of DNA replication mistakes, followed by complete dedifferentiation. In parallel, cell division stagnation reflects the degree of tumor malignancy [11]. As a result of polyploidization, cancer cells can: (i) initiate metastasis; (ii) survive “lethal” drugs; or (iii) divide asymmetrically to generate cell multilineages with higher therapy resistance [39,43]. The giant cell cycle can lead to tumor evolution, numbness, resistance, recurrence, and regeneration, as shown in Figure 1.

Genotoxic agents used in cancer therapy are associated with the induction of cell stress, ploidy, and cell size variation; these can lead to the generation of 3D structures similar to a blastocyst [44,45]. These blastocyst-like multicellular structures with a reprogrammed genome can generate resistant daughter-cells of different lineages that can acquire the capacity to go through embryonic latency, a reversible state of numbness, to survive environmental stress factors [44,46].

PGCCs can rejuvenate by way of amitotic division, generating cancer stem cells like blastomeres, that may transdifferentiate into different lineages, while the mother cell retains undifferentiated cancer properties [45].

### 3.2. PGCCs Functionalities: Plasticity, Metabolism and Resistance to Therapy

The diversity of PGCCs functions suggests polyploidy as an evolutionary source of tissue regeneration and plasticity, being explored by cancer cells to promote survival [47] and stress response [29,48], recap embryonic stages [49], exhibit adaptive exploration of multiple gene regulatory networks [48,50], and regulate biochemical and biophysical pathways [51,52].

The Warburg effect is the utilization of aerobic glycolysis for ATP synthesis, despite having abundant oxygen available. PGCCs probably use a Warburg effect similar to the embryo in state of pre-implantation by means of glucose repression of oxidative phosphorylation, as seen in the yeast Crabtree effect. The occurrence of polyploidy or aneuploidy in PGCCs ends up passing through gene losses that could alter the relative amount of enzymes favoring glycolysis, and, consequently, investing in rapid cell division [47,52].

The functionalities developed by the PGCCs also influence tumor resistance and recurrence; these are still big challenges for modern therapies, especially because they can produce stem cells able to repopulate the tumor microenvironment [53].

Thus, polyploid tumor cells can be used as a molecular model for better understanding fundamental evolutive paradigms [54,55]; evolution studies may help us answer questions about cancer resistance and success [56].

### 3.3. PGCC’s Role in Tumor Evolution

Tumor evolution is a process by which tumor cells change across time, acquiring advantageous characteristics for their survival and perpetuation, surviving immune attack, drug treatments, and diverse anti-proliferative signals [57,58]. Key mechanisms and processes altered from tumor initiation to progression include proliferation, motility, metabolism, autocrine signaling, activated intracellular pathways, inflammation, plasticity, senescence, transdifferentiation, and cell cannibalism [58].

PGCCs also share features of the Mendel and McClintock models [46], showing independent segregation for different characteristics. Stressed cells respond by redefining their genomic structure to promote tumor success [46], primarily because PGCCs exhibit diverse gene expression, which leads to neosis [48]. The stress previously suffered directs tumor cells to certain paths, including apoptosis, necrosis, senescence, mitotic catastrophe, or a mixture of these processes [48]. However, when the tumor overcomes the death threshold through alterations in regulatory networks [59,60,61], polyploidization becomes possible in a generative and reversible process [48].

**Figure 2:**
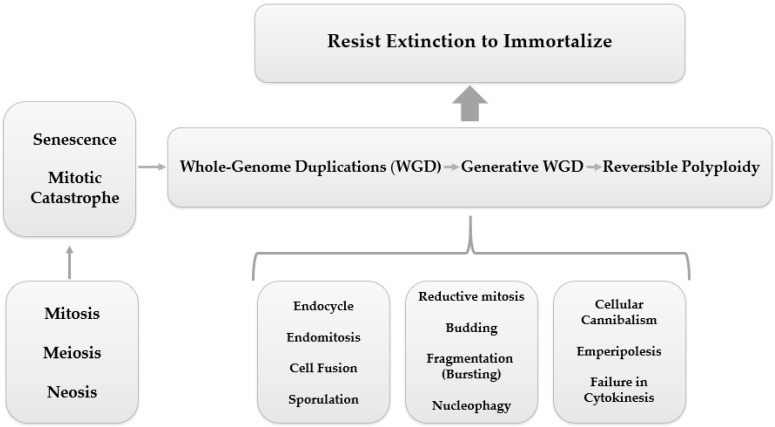
PGCC mediated tumor evolution. Cell division errors, such as mitotic arrest, can cause whole-genome duplications (WGD), which is represented by a reversible polyploidy, originated from biological processes such as endocycling, endomitosis, cell fusion, cellular cannibalism, emperipolesis, cytokinesis failure, reductive mitosis, budding, fragmentation (bursting), nucleophagy, and sporulation. Reversible polyploidy may create resistance to tumor extinction. Terms are shown in Box A1 (Appendix A).

In parallel to this mechanism of ploidy regulation, numerous other processes are shared by PGCCs, including endoreplication (endocycle and endomitosis), cell fusion, cytokinesis failure, cell cannibalism (entosis), emperipolesis, reductive mitosis, budding, fragmentation, nucleophagy, and sporulation (asexual reproduction), leading to a quite unlimited potential for PGCCs to resist extinction and achieve immortalization [48,62], as shown in Figure 2.

Vladimir Niculescu has also highlighted the potential of “pre-existing PGCCs” (before response to therapies) that can turn into multinucleated genome repair structures (MGRNs) and, subsequently, originate PGCCs [38,63,64].

### 3.4. Genome Chaos

**Figure 3:**
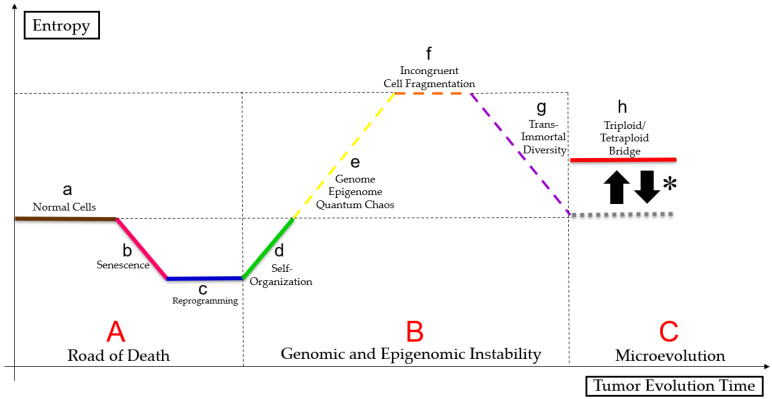
Paradoxical cancer life cycle. Normal cells (a) follow a linear ontogenetic process towards cell death interspersed with senescence (b), in a pathway called “road of death” (**A**). Some tumor cells may undergo reprogramming (c) and self-organization (d) either by mitotic catastrophe or by neosis or by mitotic slippage, because of genome, epigenome and quantum chaos (e), resulting in changes in cell fate. Tumor undergoes a macroevolutionary process (genomic and epigenomic instability (**B**)). Polyploid tumor cells undergo epigenetic and genetic regression, causing incongruent cell fragmentation (f), to achieve trans-immortal diversity (access to immortalization capacity, resistance to extinction and heterogeneous profile in the formation of multilineages of more apt tumor aneuploid cells) (g) new tumor cells follow a Darwinian evolution (microevolution (**C**)) and maintain a “triploid/tetraploid bridge” (*), between states 2n and pn–“triploid/tetraploid bridge” (h). Terms shown in Box A1 (Appendix A).

Liu [29] suggests that cell size regulation may control micro- and macroevolution, as somatic cells can follow the Waddington epigenetic landscape. PGCCs can overcome stress signals by rapidly adapting, living close to the genome, epigenome, and quantum chaos, altering cell fates, reprogramming, phylogenetically regressing, and senescing [48,65,66]. Therefore, that clonal selection enables better-adapted tumor cell survival, as shown in the Figure 3 (figure based on a bibliographic compilation by Erenpreisa and Cragg [51], Erenpreisa et al. [52], Heng and Heng [67], Heng and Heng [68], Heng and Heng [69], Liu et al. [70], Baramiya and Baranov [71] and Baramiya et al. [72]).

### 3.5. PGCCs Characterization in Diverse Cancer Types

Experimental studies with tumor cell lines suggest that targeted PGCCs therapies in cancer may be more efficient than conventional therapies. Even under similar stresses, PGCCs may exhibit tumor specific features and responses, which are shown below according to tumor type:

#### 3.5.1. Breast and Ovarian Cancer

Lin et al. [23] showed that chemotherapy-originated PGCCs upregulated the expression of Zinc finger E-box binding homeobox 1 (ZEB1) gene, correlating with metastases. Liu et al. [73] and Wang et al. [74], researching PGCCs in breast and ovarian cancer cell lines, identified a relationship between metastasis from resistant PGCCs offspring and higher lymph node grades, as well as an association between asymmetric divisions and tumor resistance.

#### 3.5.2. Colorectal Cancer

Lopez-Sánchez et al. [75], Zhang et al. [21], Fei et al. [15], Fei et al. [76], Fei et al. [77], Fei et al. [78], and Fei et al. [6] highlighted that, in colorectal cancer cell lines, PGCCs were associated with overexpression of HIF-1α, CK7, cathepsin B, E-cadherin, fibronectin, Snail, Slug, Twist-1, Syncytin 1, CD9, CD47, JNK1, cyclin B1, S100A4, and downregulation of CDC25A, CDC25B, and CDC25C, affecting cell cycle regulation, vasculogenic mimicry, migration, and invasion.

#### 3.5.3. Glioblastoma

Qu et al. [7] and Liu et al. [79], studying glioblastoma cell lines, observed a relationship between PGCCs’ number and higher tumor grade, hypoxia, red cytoplasmic inclusions, budding vasculogenic mimicry, tumor immunosuppressive microenvironment, and more aggressive phenotypes.

#### 3.5.4. Lung Cancer

Tagal and Roth [14] and Glassmann et al. [5] showed an association between staurosporine generated PGCCs and polyploid and multinucleated growth traits in lung cancer cell lines.

#### 3.5.5. Prostate Cancer and Melanoma

White-Gilbertson et al. [25,80], working with prostate cancer and melanoma cell lines, found that ASAH1 inhibition and cholesterol regulation are associated with PGCCs generation, being a bridge to tumor survival.

#### 3.5.6. Only Ovarian Cancer

Zhang et al. [81], Lv et al. [82], Zhang et al. [83], Zhang et al. [84], Niu et al. [1], Niu, Mercado-Uribe, and Liu [49], and Liu et al. [85] revealed associations between PGCCs and cell cycle, motility, metabolism, vasculogenic mimetism, auto-renovation, nuclear fragmentation, and tumor aggressiveness using ovarian cancer cell lines.

#### 3.5.7. Only Breast Cancer

Zhang, Mercado-Uribe, and Liu [13], Fei et al. [3], and Sirois et al. [24], studying breast cancer cell lines, suggested that PGCCs offspring can form organotypic structures in vitro, stromal transdifferentiation, chemoresistant cells, higher migratory capacity, and metabolic reprogramming.

Ultimately, the primary purposes of tumor polyploidization are to activate survival and aggressiveness (metastasis).

### 3.6. Autophagy, Senescence and PGCCs

Autophagy seems to be involved in the generation of PGCCs, since autophagy inhibitors before chemotherapy decreases the formation of PGCCs [86]. This process modulates PGCCs colony formation by stoppage, representing paradoxical roles both beneficial and harmful to PGCCs.

Senescence and PGCCs have been related through comparative transcriptome studies, revealing the altered expression of meiotic cell cycle genes, spermatogenesis, and EMT [87,88]. Senescence induced by chemotherapy can halter tumor proliferation, but this response also causes polyploidization, leading to PGCCs formation [89,90].

Cellular damage triggered by genotoxic stress induces senescence which, under the influence of senescence-associated secretory phenotype (SASP) and immune response evasion, facilitates reversible polyploidy through mitotic slippage, circumventing mitotic catastrophe and terminal senescence, followed by endoreplication and damage repair; this, in turn, directs new polyploid tumor cells to generate aneuploid offspring [45,50]. There mechanisms are still little understood, but they result in more aggressive tumor profiles [91,92].

## 4. Reaching New Paths

Histological, physiological, and morphological PGCCs studies used (i) CoCl_2_ and traditional cancer treatments for PGCCs induction [75,91]; (ii) classic tumor cell lineages [92,93]; (iii) PGCCs generated by budding [94,95]; and (iv) protein expression studies [96], showing that PGCCs’ acquired features are similar to classic tumor marks, described by Hanahan and Weinberg [97], Hanahan and Weinberg [98], and Hanahan [99], contributing to needed cancer traits for utter success [93].

The complexity of PGCCs motivated recent computational approaches, providing new insights into essential questions about such cells. Single cell [100], multi-omics (genomics, transcriptomics, proteomics, metabolomics, electroma) [101,102], bioinformatics, NGS [103], Big Data, and Artificial Intelligence [104], together with laboratory studies, have provided a better interpretation of polyploidy, elucidating its multiple cellular states [100], epigenetic and genetic profiles [101], spatial distributions [102], microenvironmental interactions [103], and translational advances [103,104].

## 5. Computational Perspectives

### 5.1. Bioinformatics/Computational Studies in Oncology

Modern oncology studies are using highly complex computational tools to improve data interpretation [105,106]. Furthermore, with the increase in evidence related to the role of the interaction between genes and proteins in tumor mechanisms, there was a need to integrate a new concept of medicine based on computational language to better explore all the processes involved in the emergence of cancer [107,108].

High-performance software is now used in genomics, proteomics, cell biology, physiology, pathology, therapeutics, clinical trials, and epidemiology [108]; multi-omic data processing still needs to translate in silico findings into in vivo scenarios, and is a future tool for precision medicine [107,108].

Bioinformatics also reduced the time from lab experiments to clinical oncology studies [106]. Computational biology provided a reduction in the time required to extract information due to the rapid growth of oncological data made available on online servers [106]. In this way, the increasing number of computational tools specialized in cancer has been associated with more detailed approaches to working with data [108,109]. In view of this, specialized bioinformatics in cancer are expected to play a central role in advances in translational oncology studies [106].

Novel Precision Medicine therapies, such as BRAF V600E inhibitors in patients with melanoma and PD1/PD-L1 immunotherapy for melanoma, lung, kidney, and other types of cancer [109,110], are enabling more efficient treatment with minimized side effects. In the following years, with the evolution of translational bioinformatics, advances in data sharing and integration are expected [111], improving collaborative and multidisciplinary teams [112], advances in data mining [113], expanding the ability to generate and analyze data [114], and planning therapies through better diagnosis [115] with the identification of new biomarkers and drug targets [116,117].

Artificial intelligence in oncology can refine, integrate, classify, and guide modeling prediction, classification, and screening of molecules with potential use in cancer treatment [118]. In this way, it is worth highlighting numerous other studies that express the application of computing in oncology, as shown in Table 1.

### 5.2. Computational Studies about Polyploidization and PGCCs

Bioinformatics in oncology has highlighted new ways to elucidate the role of chromosomal aberrations, cell cycle errors, intratumoral heterogeneity, and tumor evolution in the face of new, more aggressive cancer phenotypes [131]. In parallel, multimodal, multi-omics, pan-cancer, and single cell analyses integrate data from diverse biological systems, clarifying tumor peculiarities, as for polyploidy [132].

Computational tools have been tested for different purposes to study cancer and polyploidy [133,134]. Among these approaches, we would like to highlight studies on nucleotide polymorphisms (SNPs), structural variants (SVs), unique somatic tumor-specific variants [135], genome annotation, mutation detection, evolutionary analysis, gene function, comparative genomics [136,137], identification of haplotypes, subgenomes, and assembly of whole genomes [137].

Among the computational approaches to tumor polyploidy, Anatskaya et al. [138] built a protein-protein interaction network (PPIs) of bivalent genes using the STRING server to investigate the regulation of polyploidy-associated gene expression, suggesting a role for polyploidy in the upregulation of oncogenes and downregulation of tumor suppressor genes. Furthermore, down-regulation of the DNA damage response (DDR) has been shown to increase damage tolerance and inhibit apoptosis.

Furthermore, Czarnecka-Herok et al. [88] performed gene enrichment analysis (GSEA) in doxorubicin-induced senescent/polyploid HCT116 and MCF-7 cell lines. In that study, an enrichment analysis revealed changes in genes involved in controlling meiosis and mitosis. Interestingly, enrichment of spermatogenesis genes was also observed, supporting the premise that genetic alterations in tumors may be associated with germline expression of normally silenced genes, a characteristic that may be related to tumor malignancy.

Additionally, Yang et al. [139] used GEO2R software, genetic ontology (GO), and the Kyoto Encyclopedia of Genes and Genomes (KEGG) to analyze databases for integrated annotation, visualization, and discovery (DAVID) of differentially expressed genes in cervical cancer cells using Affymetrix Human Genome arrays. In addition, they used the STRING software to build protein-protein interaction (PPI) and to analyze differentially expressed genes involved in cancer prognosis, with the help of UNLCAN and Oncomine browsers. In total, 57 differentially expressed genes were recognized, especially those enriched in the mitotic cell cycle G1/S transition, participating in cytokine-cytokine receptor interaction, including WD Repeat and HMG-Box DNA Binding Protein 1 (WDHD1), a gene involved in cancer promotion and polyploidy induction.

Moreover, using the Gene Expression Omnibus (GEO) database, Yan et al. [140] analyzed different gene expression profiles (GSE54238 and GSE84004) in the development of hepatocellular carcinoma from data taken from GPL16955 and GPL22109. STRING and Cytoscape software were used to visualize the integrated regulatory networks and raw data were analyzed using the multi-array averaging algorithm in the R Affy package. The PPI network they constructed highlighted five main core genes with the highest degree of connectivity, including *AURKA*. This gene showed a role in regulating the mitosis G2/M transition and its overexpression is associated with polyploidy and genomic instability.

In addition, Wang et al. [141] investigated the variation of GSE38241, GSE69223, GSE46602, and GSE104749 in the development of prostate cancer by using the GPL570 Affymetrix Human Genome U133 Plus 2.0 Array and GPL4133 Agilent-014850 Whole Human Genome microarray 4 × 44 K G4112F platforms. For this purpose, GEO software was used. Furthermore, a PPI network was also built using STRING and MCODE and the enrichment of the GO function was conducted with the DAVID software. The study revealed 20 genes related to mitosis and cell division and the process of carcinogenesis, including cyclin B1 (CCNB1). Elevated levels of cyclin B1 have been associated with polyploid cell uptake and shown to be a potential marker for diagnosing prostate cancer.

Another comparative study using bioinformatics for transcriptome analysis of polyploid versus diploid cells from normal mammalian tissues highlighted a potential downregulation of circadian clock genes by c-Myc (a cell cycle regulator), whereas polyploidization correlated with deletion of bivalent genes responsible for circadian rhythm regulation [142].

Structural variants (SVs) are crucial to understanding cancer polyploidy. Kosugi et al. [143] evaluated the performance of 69 SV detection algorithms using whole exome sequencing (WES) datasets, using the GRIDSS, Lumpy, SVseq2, SoftSV, Manta, and Wham algorithms to improve variant detection accuracy, focusing on SV size. This means that careful selection of algorithms for each type and size range of SVs is crucial for accurate SV detection. Therefore, it results in the most accurate information possible regarding the correlation between tumor structural alterations and polyploidization.

In addition to such basic research applications, computing linked to tumor polyploidy also highlights new paths for clinical studies and strengthened by the advancement of high-throughput technology integration. Among the main computational approaches, machine learning algorithms stand out [144], showing promise for the evolution of translational research in oncology and initiating a revolution in tumor mechanistic knowledge [145].

In a search on polyploidy clinical trials around the world at the website https://clinicaltrials.gov (accessed on 15 February 2023), only two studies were found for the terms “cancer” and “polyploidy”. The distribution of clinical trials around the globe demonstrated a study recruiting patients (USA) and another already completed (Germany), confirming that research on PGCCs needs to be further developed worldwide.

Although there are still obstacles to the implementation of computational PGCC analyses in clinical practice, some tools demonstrate new trends towards faster and more personalized diagnoses and treatments [146]. In addition, an expansion of PGCCs computational models will provide valuable data for the understanding of this complex phenotype and its translational relevance in cancer [147].

Multiple analyses, including multi-omics, comparative phylostratigraphy, mathematical modeling, and others, will provide a better understanding of cell subtype and state. Table 2 demonstrates the impact of computation in the area of tumor polyploidy.

The computational era has provided simulation tools to better understand cell-to-cell interactions in normal and altered cell cycles, relying on specific and detailed cell cycle models, with the aim of understanding the dynamics of tumor formation and the potential outcomes associated with polyploidy [161]. Such innovations and improvements in oncology are only possible through the integration and multidisciplinarity of research groups.

As highlighted in this review, advances in computing permeate artificial intelligence approaches, machine learning algorithms [162], personalized targeting [163], and several other approaches. Broad access to low cost computational tools will expand specialized computational models in the study of cancer and PGCCs [162,163].

### 5.3. Future Directions

Applications of systems biology, computational biology, and bioinformatics, as shown by the studies in Table 1 and Table 2, allowed the analysis of high-throughput samples through artificial intelligence, machine learning, and other techniques [162,163], resulting in data about the identification of genetic signatures [164], new biomarkers [163,165], multiscale modeling [164,165], and visualization and interpretation of cellular data [164].

Research with artificial intelligence will embody the personalization of prevention, diagnosis, and therapy [162,166]. New biotechnological approaches will provide better cancer treatment, especially in cases of resistance, numbness, metastasis, and recurrence correlated to the polyploidization process. Molecular computers inside tumor cells acting as sensors, digital pathology, personalized biopsies, analysis of microscopic images and exams, synthesis of biological structures, and pharmacological bioassays will increase the computational area as required to solve demands in these complex studies [161,162,167].

**Figure 4:**
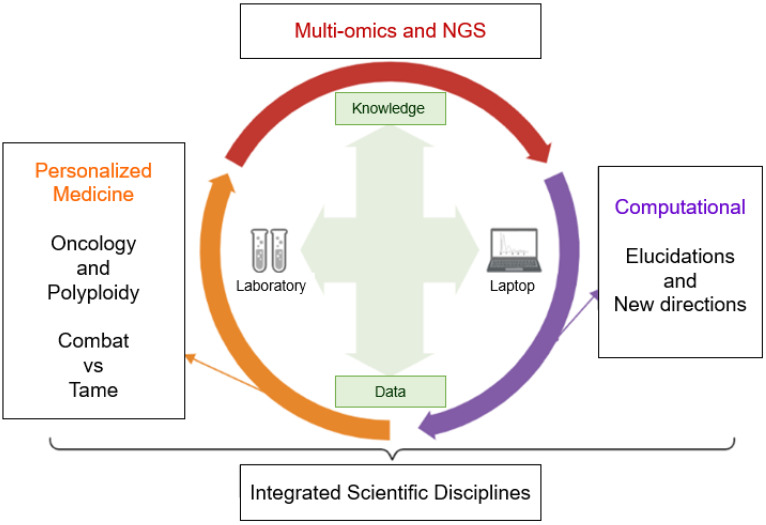
Integrated scheme of computational resources in oncology and polyploidy. The biological computational era has provided numerous advances on the amount of biological data and their significance. Crosstalk between computing and research laboratory is needed to create personalized medicine through multi-omics. Note: created with BioRender.com (accessed on 15 March 2023).

In addition, machine learning will provide new answers for studies in oncology and tumor polyploidy (especially for the understanding of PGCCs), and will further extend its sophisticated approaches with studies in microrobotics for cell manipulation [168,169], ploidy inference based on co-sequencing of DNA and RNA in individual nuclei [169], multi-omics [170,171], NGS [170], single-cell [171], and other approaches capable of bringing new paths to the understanding of polyploidization in cancer (as indicates Figure 4).

Computational tools provide ideal support for understanding self-organization in face of cellular chaos, linked to the complex genomic functioning of the polyploid cells [172,173]. Single-cell RNA sequencing approaches will increasingly add new light to polyploidy, as shown in studies on hypertranscription [164,172,174]. In the long term, an increasingly prominent role for bioinformatics in elucidating polyploidy in tumors is expected. To achieve this goal, scientists from different fields are expected to come together to understand, tame, and fight cancer [172,173].

## 6. Conclusions

Polyploidization in tumors runs through multiple mechanisms phylogenetically shared between unicellular and multicellular organisms, resulting in evolutionary advantages for the tumor, thus reverberating an intrinsic complexity that is highly difficult to access through isolated studies. Thus, the need for integrated and multidisciplinary approaches capable of elucidating polyploidy in tumors becomes clear. Computational biology, bioinformatics, NGS, single-cell analysis, multi-omics studies, and systems biology reveal a growing and promising potential in the understanding of polyploidization as well as its innovative potentials to create personalized, comprehensive, and multifactorial therapies in cancer treatment.

## Figures and Tables

**Table 1:** Computational studies in oncology.

Cancer Type	Study Description	Author
Breast Cancer	Computational studies about differentially expressed genes, omics, and systems biology data provided the identification of new gene signatures-*NUSAP1*, *MELK*, *CENPF*, *TOP2A*, and *PPARG*-genes related to chemoresistance, potential biomarkers, and new therapeutic targets associated with tumor polyploidy.	Alam et al. [119], Kaur et al. [120], Mukherjee et al. [121], Yadav et al. [122], and Yang et al. [123]
Prostate Cancer	Systems biology and bioinformatics identification of key genes as biomarkers of diagnostics, prognosis, and treatment (*EGFR*, *MYC*, *VEGFA*, *PTEN*).	Khan et al. [124]
Melanoma	Mathematical modeling after therapeutic regression was able to identify a triphasic signaling pathway in tumor regression.	Kumari et al. [125]
Pan-cancer	Identified the relationship between *SLC7A11* expression and tumor microenvironment, using bioinformatics in 20 tumors.	Lin et al. [126]
Colorectal and Uterine Cancer	Found potential genes and their signaling pathways using bioinformatics and systems biology.	Nguyen et al. [127] andReza et al. [128]
Lung Cancer	An integrated systems biology and bioinformatics approach provided the detection of genetic correlations between COVID-19 and small cell lung cancer and the interaction of biological pathways associated with tumor polyploidization.	Roudi et al. [129] and Zhuang et al. [130]

**Table 2:** Computational biology applied to polyploidy studies.

Cancer Type	Study Description and Key Findings	Author
Ovary Cancer	Computational research based on NGS, total RNA, and microarray sequencing, using primary tumors, cell lines, and tumor chemoresistance highlighted the association with tumor polyploidy.	Adibi, Moein and Gheisari [148], Quinton et al. [149], Rohnalter et al. [150]
Lung Cancer	Investigated the role of natural and synthetic mutations in tumor migration and invasion.	Alwash et al. [151]
Several types of cancer	Molecular mechanisms associated with polyploidy, cell plasticity, unicellularity, energy metabolism, tumor DNA damage in tumors, phylogenetic approaches, and molecular modeling were used study the effects of PGCCs on gene expression, tumor microenvironment, and p53.	Anatskaya and Vinogradov [138], Anatskaya et al. [152], Anatskaya et al. [142], Kimmel et al. [153], and Potapova et al. [154]
Breast Cancer	Studied formation of PGCCs by mechanical stress.In silico studies to detect gene signatures related to PGCC formation.	Buehler et al. [155] and Rantala et al. [156]
Colorectal Cancer	*S100A10* expression changes caused by differential SUMOylation during the migration of PGCCs.	Fu et al. [157] and Zhao et al. [158]
Gastric Cancer	MiRNA sequencing to study the role of epigenetic in regulation of Aurora kinase A (AURKA) expression.	Gomaa et al. [159]
Cervical cancer, Breast Cancer and Burkitt Lymphoma	In silico studies using Mitelman’s database to uncover PGCC’s role in DNA repair, genetic variation, and tumor survival.	Salmina et al. [160]
Prostate Cancer and Melanoma	Transcriptome analysis of PGCCs after *ASAH1* treatment leading to cholesterol metabolism alterations.	White-Gilbertson et al. [80]
Nasopharyngeal Cancer	Induction of PGCC by autophagy.	You et al. [86]

## Data Availability

Not applicable.

## Abbreviations

ASAH1	N-acylsphingosine amidohydrolase (acid ceramidase) 1
ATP	Adenosine triphosphate
AURKA	Aurora kinase A
BRAF V600E	Mutation of the BRAF gene
CD47	Integrin-associated protein
CD9	Motility-Related Protein
CDC25A	M-phase inducer phosphatase 1
CDC25B	M-phase inducer phosphatase 2
CDC25C	M-phase inducer phosphatase 3
CENPF	Centromere Protein F
CK7	Cytokeratin 7
CoCl2	Cobalt chloride
CSC	Cancer stem cells
EGFR	Epidermal growth factor receptor
EMT	Epithelial-Mesenchymal Transition
ERK	Extracellular signal-regulated kinase
EZH2	Enhancer of zeste homolog 2
HIF-1α	Hypoxia-inducible factor-1
JNK	c-Jun N-terminal kinase
JNK1	c-Jun N-terminal kinase 1
LOH	Loss of heterozygosity
MELK	Maternal embryonic leucine zipper kinase
MYC	MYC Proto-Oncogene
NUSAP1	Nucleolar and spindle associated protein 1
p38MAPK	p38 mitogen-activated protein kinase
PACCs	Poly-aneuploid cancer cells
PD1/PD-L1	Programmed cell death protein-1
PGCCs	Polyploid giant cancer cells
PPARG	Peroxisome proliferator activated receptor γ
PTEN	Phosphatase and tensin homologue
S100A4	S100 calcium binding protein A4
*SKP2*	S-phase kinase associated protein 2
SLC7A11	solute carrier family 7 member 11
Slug	Snail family transcriptional repressor 2
Snail	Snail family transcriptional repressor 1
*SSP*	Staurosporine
STC1	Stanniocalcin-1
TNBC	Triple negative breast cancer
TOP2A	DNA Topoisomerase II α
TPL	Triptolide
Twist-1	Twist family bHLH transcription factor 1
VEGFA	Vascular endothelial growth factor A
VM	Vasculogenic mimicry
WGDs	Whole-genome duplications
ZEB1	Zinc finger E-box binding homeobox 1

## Appendix A

Box A1 Important terms and definitions.  **Aneuploidy:** autosomal abnormality that occurs mainly during meiosis and affects the number of chromosomes.  **Bivalent chromatin:** segments of DNA linked to histone proteins that have epigenetic regulators repressors and activators in the same region. These regulators work to increase or silence gene expression.  **Endocycle/Endocycling:** nuclear genome replication in the absence of mitosis, which leads to high nuclear genetic content and polyploidy.  **Endomitosis:** chromosome duplication within the intact nuclear membrane, without telophase or cytokinesis, resulting in a single cell with twice the chromosomal content.  **Endoreplication/Endoreduplication/Endopolyploidy** (Mitotic Cycle variant forms): increased number of chromosomes in cells.  **Euploidy:** numerical chromosomal variation affecting the entire chromosomal set (n).  **Incongruous partition:** interconversion phase between ploidy and mitosis cycles capable of inducing depolyploidization.  **Ploidy:** complete numerical set of chromosomes in a cell (n).  **Progressive Partial Endoreduplication/DNA Overreplication:** process of almost perfect linearity between DNA content and the number of endoreduplication cycles.  **Transcendent Regression:** ability to recover primordial phylogenetic features due to deep homology between multicellular and unicellular organisms ((formation of PGCCs, polyaneuploid cancer cells (PACCs), multinucleated tumor cells/multinucleated genome repair structures (MNGCs), micronucleated tumor cells, cells tumor hybrids (cell fusion) and supergiant “nurse” cells, following the amoeba-cancer model and the cancer life cycle)).  **Trans-Immortal Diversity:** access to immortalization capacity (sustained by a constant renewal of telomerase function/Hayflick limit renewal), resistance to extinction and heterogeneous profile in the formation of multilineages of more apt tumor aneuploid cells after a process of depolyploidization (after ploidy cycle).  **Types of endopolyploidy:** endocycles, endomitosis and progressive partial endoreduplication.
